# Xinkeshu Improves Endothelial Function and Augments Reendothelialization Capacity in Coronary Artery Disease with Anxiety/Depression

**DOI:** 10.1155/2021/5561272

**Published:** 2021-07-18

**Authors:** Jiapan Sun, Meiling Zhou, Guanghui Lv, Wenling Li, Yuanya Liu, Jiawen Liang, Jianning Zhang, Shijun Zhang, Yuanfei Deng, Jun Tao

**Affiliations:** ^1^Department of Geriatrics, Peking University Shenzhen Hospital, Shenzhen Peking University-The Hong Kong University of Science and Technology Medical Center, Shenzhen, Guangdong, China; ^2^Department of Hypertension and Vascular Disease, The First Affiliated Hospital, Sun Yat-Sen University, Guangzhou, Guangdong, China; ^3^Department of Traditional Chinese Medicine, The First Affiliated Hospital, Sun Yat-sen University, Guangzhou, Guangdong, China

## Abstract

The disruption of endothelial homeostasis is the hallmark of coronary artery disease (CAD) and psychological disorders such as anxiety/depression. Xinkeshu (XKS), a traditional Chinese patent medicine, plays an essential role in CAD and psychological condition; however, the mechanisms underlying the effects of XKS on the endothelial function and endogenous endothelium-repair capacity in CAD patients with anxiety/depression remain elusive. In this study, endothelial function and endothelial progenitor cell- (EPC-) mediated reendothelialization capacity were compared among age-matched healthy subjects, CAD patients with or without anxiety/depression. Besides, CAD patients with anxiety/depression received 1-month XKS treatment. Anxiety/depression symptoms were evaluated by Generalized Anxiety Disorder 7-item (GAD-7)/Patient Health Questionnaire-9 (PHQ-9) score, endothelial function was tested by flow mediated dilation (FMD) measurement, and EPC-mediated reendothelialization capacity was evaluated by a carotid artery injury model in nude mouse (*n* = 6) with the injection of XKS-incubated EPCs from CAD patients with anxiety/depression. The results showed that FMD and EPC-mediated reendothelialization capacity of CAD patients with anxiety/depression were compromised compared to healthy subjects and CAD patients without anxiety/depression. After 1 month of XKS treatment, FMD increased from 4.29 ± 1.65 to 4.87 ± 1.58% (*P* < 0.05) in CAD patients with anxiety/depression, whereas it remained unchanged in the controls. Moreover, XKS decreased GAD-7 and PHQ-9 scores. Meanwhile, incubating XKS enhanced *in vivo* reendothelialization capacity and *in vitro* apoptosis of EPCs from CAD patients with anxiety/depression, which was associated with the upregulation of CXC-chemokine receptor 7 (CXCR7) and inhibition of phosphorylation of p38 signaling. CXCR7 knockdown abolished the beneficial effects of XKS, which was rescued by p38 inhibitor SB203580. Our data demonstrate for the first time that XKS improves endothelial function and enhances EPC-mediated reendothelialization through CXCR7/p38/cleaved casepase-3 signaling and provides novel insight into the detailed mechanism of XKS in maintaining endothelial homeostasis in CAD patients with anxiety/depression.

## 1. Introduction

Coronary artery disease (CAD) combined with anxiety and depression accelerates the process and aggravates the prognosis of cardiovascular disease [1, 2]. The prevalence of anxiety and depression is increasing among CAD patients [3–5], and it has been reported to be associated with raised cardiovascular mortality [6–8]. Emerging evidence suggests that CAD and anxiety/depression share a crucial pathophysiological characteristic—the disequilibrium of endothelial homeostasis [9, 10]. Thus, improving endothelial function in CAD patients with anxiety/depression has gained immense attention nowadays.

Previous studies demonstrate that flow-mediated dilation (FMD) can predict the occurrence of adverse cardiovascular outcomes and prognosis of cardiovascular diseases [11]. Simultaneously, circulating endothelial progenitor cells (EPCs), a group of precursor cells derived from the bone marrow, was found to play an essential role in maintaining endothelial integrity, representing the capacity of endogenous endothelial repairment [12, 13]. Studies showed that the decline in the function and number of circulating EPCs was associated with adverse clinical outcomes in CAD individuals [14, 15], and decreased brachial arterial FMD was also observed in such patients with elevated depression or stress score [14]. Based on these, both of FMD and circulating EPC-mediated reendothelialization capacity were considered classical indexes to reflect the endothelial homeostasis.

Notably, the holistic therapeutic effect of traditional Chinese medicine (TCM) on CAD and psychological disorders has also been recognized in recent years. Xinkeshu (XKS), a traditional Chinese patent medicine comprising five commonly used Chinese herbs: roots of *Salviae miltiorrhizae* Bunge (Dan-Shen), roots of *Pueraria lobata* (Willd) Ohwi (Ge-Gen), roots of *Panax notoginseng* (Burk) F. H. Chen. (San-Qi), fruit of the *Crataegus pinnati* de Bunge (Shan-Zha), and roots of *Radix Aucklandiae* (Mu-Xiang), is widely used in the treatment of CAD. Importantly, previous studies have reported that XKS protects the endothelium in rats through improving endothelial function, reducing blood viscosity, and inhibiting inflammation [16]. Clinical trial demonstrates that XKS can effectively improve anxiety and depression symptoms in patients with CAD [17]; however, the mechanisms underlying the effect of XKS on endothelial function and endothelium-reparative capacity remain elusive in such individuals.

Accumulating evidence indicates that chemokine receptors are highly involved in the modulation of EPC-mediated endothelial repair [18]. CXC chemokine receptor seven (CXCR7) has been identified as a novel chemokine receptor response to stromal cell-derived factor 1 (SDF-1) [19], which plays an essential role in the vascular system including vascular formation and protection, endothelial cell growth, and survival [20]. Studies show that CXCR7 mediates the endothelial adhesion and antiapoptosis of renal progenitor cells, which promote renal progenitor cells to repair necrosis of kidney tissue [21]. P38MAPK is also a key factor in migration, proliferation, and metabolism of different cells [22, 23]. Previous studies have reported that the CXCR7/p38 pathway plays a pivotal role in regulating the homing and apoptosis of EPCs and repair of endothelial injury [24]. However, whether XKS protects the endothelium through CXCR7/p38MAPK/casepase-3 signaling-mediated apoptosis and vascular endothelial repair capacity of EPCs in CAD patients with anxiety/depression remains to be uncovered.

To test our hypothesis, we compared the endothelial function and endothelial progenitor cell- (EPC-) mediated reendothelialization capacity among age-matched healthy subjects, CAD patients with or without anxiety/depression at first. Then, CAD patients with anxiety/depression received XKS treatment to verify whether XKS could restore endothelial function and ameliorate anxiety/depression symptoms in CAD patients with anxiety/depression. Meanwhile, we explored XKS's effect on EPC-mediated reendothelialization capacity and the relation with CXCR7/p38/cleaved caspase-3 signaling in vitro. This study may provide a novel insight to the beneficial effects of XKS on endothelial homeostasis in CAD patients with anxiety/depression.

## 2. Methods and Materials

### 2.1. Study Design and Participants

60 age- and sex-matched coronary artery disease (CAD) individuals with or without depression/anxiety with 1 : 1 ratio and 30 matched healthy individuals were included to analyze the baseline characteristics and endothelial function.

**Figure 1:**
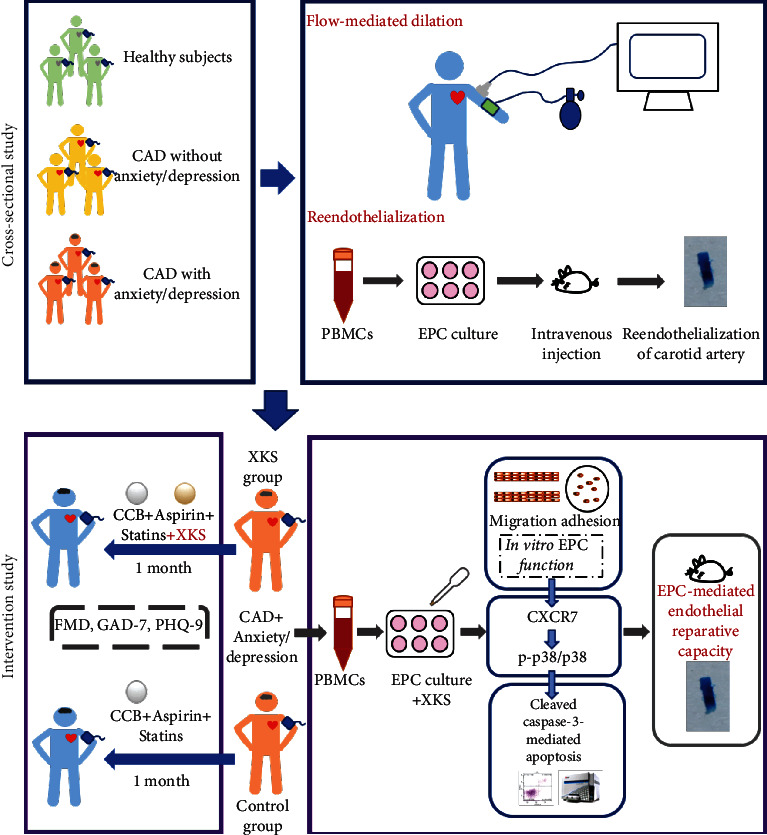
Flow chart. Patients received routine treatment combined with XKS or routine treatment for 1 month to assess the arterial function, anxiety/depressive symptoms, and the mechanisms.

For the intervention study, eligible participants were aged 18–75 years, diagnosed with CAD by coronary angiography, and their Generalized Anxiety Disorder 7-item (GAD-7)/Patient Health Questionnaire-9 (PHQ-9) score is between 5 and 14. The exclusion criteria were severe liver or kidney dysfunction or malignant tumors, cardiovascular or cerebrovascular injury in previous 3 months, pregnant women, lactation or menstruation, or allergy to any component of XKS. Thereafter, 30 patients with CAD and anxiety/depression were randomly divided into the XKS and control groups. The XKS group received routine treatment (aspirin 100 mg qd, atorvastatin 20 mg qd, and amlodipine 5 mg qd) combined with XKS tablets 37.2 g/day, tid, for 1 month [Shandong Wohua Pharmaceutical Co Ltd, CHN, Shandong, China (Supplementary Figure S1)]. The control group received routine treatment (aspirin 100 mg qd, atorvastatin 20 mg qd, and amlodipine 5 mg qd) for 1 month. Blood samples were collected, arterial function was assessed by FMD measurement, and anxiety and depressive symptoms were evaluated by using the GAD-7 and PHQ-9 scores (Figure 1, Supplementary Figure S2) at baseline and after the intervention. All subjects provided written informed consent, and ethics approval was granted by the Ethics committee of the First Affiliated Hospital, Sun Yat-sen University in Guangzhou ([2017] 154), China. The trial was registered on the ISRCTN registry (ISRCTN22691979).

### 2.2. FMD Measurement

All patients in this study underwent FMD examination of the brachial artery at baseline and after the treatment through noninvasive ultrasound scan (UNEXEF38G, Sakae, Japan), and they were fasted and had a rest at least for 10 min before the examination. The machine dynamically recorded artery diameter at baseline and reactive hyperemia period (the cuff inflated to the greater of 50 mmHg above systolic pressure or 200 mmHg for 5 min) as previously described [25]. FMD was calculated as follows: FMD (%) = (maximum diameter at rest) × 100/(diameter at rest).

### 2.3. Blood Pressure Measurement

Blood pressure was measured by an electronic sphygmomanometer (HEM-7071, OMRON, China) with the subjects resting at the sitting position at least for 10 min in a quiet room. The average of measurements obtained on at least three separate occasions was used as SBP and DBP of each subject.

### 2.4. EPC Culture and Identification

EPCs were isolated and cultured as previously described [26]. After 4 days of culture, the nonadherent cells were removed and transferred to endothelial cell basal medium-2 (EBM-2) (Lonza, Swiss). After 21–28 days of culture, late EPCs were examined via flow cytometry (Cytoflex, Beckman Coulter, USA) to identify the endothelial markers including CD31, CD34, and CD309 (BD Pharmingen), as previously described [27], and were then used for the following experiments.

### 2.5. EPC Migration In Vitro

A total of 2 × 10^4^ EPCs were resuspended in 250 *μ*L EBM-2 and pipetted into the upper section of a modified Boyden chamber (Costar Transwell® assay, 8 *μ*m pore size, Corning, NY). The chamber was placed in a 24-well culture dish containing 500 *μ*L EBM-2 supplemented with 100 ng/mL SDF-1 (Peprotech, USA). After 12 h incubation at 37°C, the transmigrated cells were fixed with 4% paraformaldehyde for 15 min and 0.3% crystal violet for another 15 min for further enumeration and analysis.

### 2.6. EPC Adhesion In Vitro

A monolayer of human umbilical vein endothelial cells (HUVECs) was prepared in a six well plate (2 × 10^5^ cells per well) and incubated for 48 h before the assay; thereafter, the HUVECs were pretreated with 1 ng/mL tumor necrosis factor-*α* (TNF-*α*, Peprotech, USA) for 12 h. Next, 1 × 10^5^ CM-Dil- (CellTracker™ CM-Dil, Invitrogen, USA) labeled EPCs were added to each well and incubated for 3 h at 37°C. Nonattached cells were gently removed with phosphate-buffered saline (PBS, Thermo Fisher Scientific, USA) twice, and adherent EPCs were fixed with 4% paraformaldehyde. Subsequently, the cells were stained with DAPI (Beyotime Biotechnology, China) stain for 10 min and then enumerated by independent investigators blinded to the treatment.

### 2.7. Real-Time Polymerase Chain Reaction

Total RNA was extracted using an mRNA abstraction kit (EZBioscience, USA). Reverse transcription was performed using a Transcript II real-time polymerase chain reaction (RT-PCR Kit; EZBioscience, USA), followed by semiquantitative RT-PCR using Bio-Rad iQ5. Data were normalized to mRNA levels of GAPDH and were analyzed by the 2^−∆∆^CT method. The final results were presented as the percentage of control. The primer CXCR7 A (sense) was 5′-TCTGCATCTCTTCGACTACTCA-3′, and the primer CXCR7 B (antisense) was 5′-GTAGAGCAGGACGCTTTTGTT-3′.

### 2.8. Western Blot

Total EPC protein was extracted and quantified using RIPA lysis buffer (Beyotime Biotechnology, China) containing protease inhibitors and BCA assay kit (Thermo Fisher Scientific, USA) separately. Protein extracts were separated via SDS-PAGE and transferred to polyvinylidene difluoride membranes (Roche, Indianapolis, IN, USA). The following antibodies were used: anti-CXCR7 antibody (1 : 1000; Affinity, USA), anti-p38 mitogen-activated protein kinase (MAPK) antibody (1 : 1000; Affinity, USA), anti-p-p38 MAPK antibody (1 : 1000; Affinity, USA), anti-cleaved casepase-3 antibody (1 : 1000; Cell Signaling Technology, USA), anti-GAPDH antibody (1 : 1000; Cell Signaling Technology, USA), and HRP-conjugated anti-rabbit IgG (1 : 3000; Cell Signaling Technology, USA). Protein bands were visualized using an ECL chemiluminescence system (Thermo Fisher Scientific, USA).

### 2.9. RNA Interference

To knock down CXCR7 expression in EPCs, specific siRNAs against human CXCR7, alone with negative control (Bioneer, Republic of Korea), were transfected into EPCs using Lipofectamine 3000 (Thermo Fisher Scientific, USA). After transfection for 48 h, the expression of CXCR7 was determined via qPCR and western blot.

### 2.10. Detection of Apoptosis

Apoptosis EPCs were detected with Annexin V-FITC/propidium iodide (PI) Apoptosis Detection Kit (BD Biosciences) after cell treatment. According to manufacturer's instructions, the cells were harvested by centrifugation (300 g/5 min), washed with cold PBS (300 g/5 min) and 1× binding buffer (300 g/5 min) once each. After that, cells were resuspended in 100 *μ*L 1× binding buffer and incubated with Annexin V-FITC (5 *μ*L) and PI (5 *μ*L) at room temperature in the dark for 15 min. The cells were added 300 *μ*L × binding buffer and tested by flow cytometry (Cytoflex, Beckman Coulter, USA). The positive cells were calculated and analyzed by FlowJo software (Tree Star Inc., Ashland, OR).

### 2.11. Wire Injury of Mouse Carotid Artery and Reendothelialization In Vivo

Wild-type male BALB/c nude mice (4–6 weeks) were collected from Sun Yat-sen University (Guangzhou, China). Mice were maintained under a 12 h light/dark cycle with free access to food and drinking water. Wire injury in the carotid artery was performed as previously described [24, 27]. Animals were anesthetized with an intraperitoneal injection of pentobarbital sodium (50 mg/kg). Considering a middle-line neck incision on the ventral side, the left common carotid artery, including the bifurcation, was exposed. A bulldog clamp was placed around the left common carotid artery proximal to the aortic arch for temporary control of blood flow. Thereafter, a 6–0 suture was placed around the left external carotid artery. Next, an incision hole was made in the left external carotid artery, and a flexible wire (0.38 mm) was introduced into the left common carotid artery and passed thrice toward and forth with rotation to denude the endothelium. The left external carotid artery was tied proximal to the incision hole after removing the wire. Furthermore, the skin incision was closed with surgical sutures. EPCs with XKS incubation or without XKS incubation (5 × 10^5^ cells) resuspended in 100 *μ*L of prewarmed PBS (37°C) were transplanted 3 h after wire injury via tail vein injection, and a similar volume of PBS was injected as a placebo control. Three days after transplantation, endothelial repair was evaluated as the reendothelialization area of the denudated endothelial zone by staining with Evans blue dye. The experiments were approved by Animal Experiment Ethics Committee of Sun Yat-sen University, Guangzhou, China ([2017] 107). All experimental protocols followed the Guide for the Care and Use of Laboratory Animals published by the US National Institutes of Health (National Institutes of Health Publication No. 85–23).

### 2.12. Hematoxylin-Eosin Staining

The carotid arteries were harvested rapidly, washed with normal saline, fixed in 4% formaldehyde, and embedded in paraffin. The paraffin blocks were cut into 2 *μ*m thick sections and stained with hematoxylin and eosin. Images were acquired using a light microscope (Olympus BX63) with 400x magnification.

### 2.13. Immunohistochemical Staining

Immunohistochemical staining was performed as described previously [28]. Briefly, cross-sections of nude mice carotid arteries were deparaffinized and rehydrated and antigen retrieval was carried out by microwave oven heating in sodium citrate buffer (0.01 mol/L, pH 6.0). Sections were incubated with rabbit monoclonal CD31 antibody (Cell Signaling Technology Inc., MA, USA) followed by HRP Anti-Rabbit DAB Detection kit (Cell Signaling Technology Inc., MA, USA) according to the manufacturers' instructions. Images were acquired using a light microscope (Olympus BX63) with 400x magnification.

### 2.14. Statistical Analysis

All results were expressed as the mean ± standard deviation (SD) and plotted using GraphPad Prism 8 software. The Shapiro-Wilk test was used to determine the normality of data and F test to assess homogeneity of variance. All data in the article passed normality and equal variance tests. Two groups of normal distribution data were analyzed using 2-tailed Student's *t*-test. Statistical significance of multiple groups was assessed by one-way analysis of variance (ANOVA) followed by Tukey's test. Biological experiment replicates in each group were specified in the figure legends (*N* value). *P* < 0.05 was considered to denote statistical significance. All statistical analyses were performed using SPSS statistical software (SPSS version 23.0).

## 3. Results

### 3.1. Significant Reduction in Endothelial Function and EPC-Mediated Endothelium-Reparative Capacity in CAD Patients with Anxiety/Depression Compared to Healthy Subjects and CAD Patients without Anxiety/Depression

**Figure 2:**
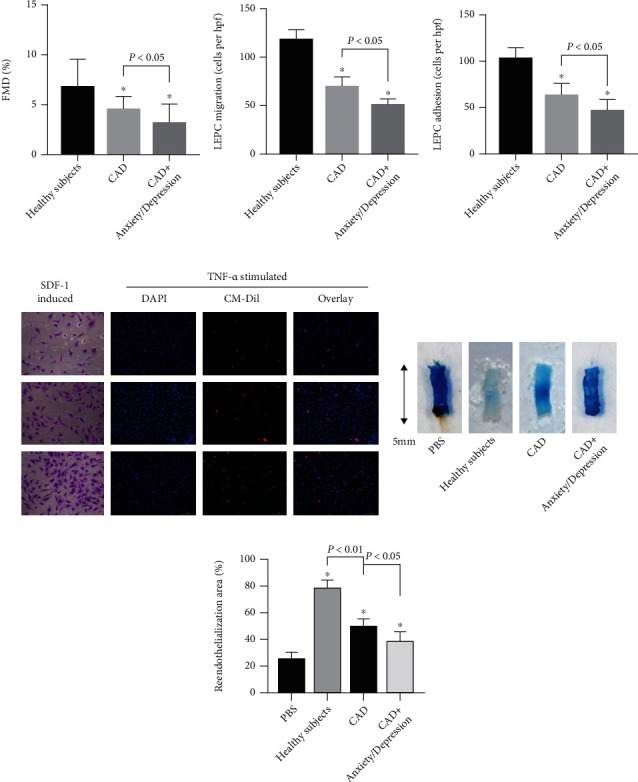
The endothelial function and endothelial repair capacity of EPCs in CAD patients with or with anxiety/depression and healthy subjects. (a) The mean value of FMD (^∗^*P* < 0.01*vs.* healthy subjects, *n* = 30). (b) The mean value of migrated cell numbers (^∗^*P* < 0.01*vs.* the XKS group, *n* = 6), (c) the mean value of adhering cell numbers (^∗^*P* < 0.01*vs.* the XKS group, *n* = 6) of EPCs, and (d) the representative photographs of migration and adhesion function. (e) Representative photographs and (f) quantification analyses of reendothelialization areas (white) of the injured carotid arteries in nude mice 3 days after carotid denudation surgery plus intravenous injection of EPCs (5 × 10^5^ cells) from CAD patients with or without anxiety/depression or healthy subjects (^∗^*P* < 0.01*vs.* healthy subjects, *n* = 6).

We evaluated the endothelial function and endothelial reparative capacity of EPCs in CAD patients with or without anxiety/depression and healthy subjects with a 1 : 1 : 1 ratio, 30 subjects in each group. As listed in Table S2, most of the baseline characteristics did not differ significantly between these three groups except for blood pressure. Compared to the other two groups, FMD was lower in CAD patients with anxiety/depression (3.27 ± 1.82% *vs.*4.64 ± 1.18% and 3.27 ± 1.82% *vs.*6.91 ± 2.62%, respectively, all *P* < 0.01; Figure 2(a)). It has been proved that EPCs play a crucial role in endogenous injured endothelial recovery, and the transplantation of EPCs could increase reendothelialization of denuded carotid arteries in nude mice [24]. However, the results show that the *in vitro* function of EPCs (*P* < 0.01, Figures 2(b)–2(d)) and *in vivo* reendothelialization capacity of EPCs from CAD patients with anxiety/depression were the worst among the EPCs from CAD patients without anxiety/depression and healthy subjects (42.3 ± 6.1% *vs.*55.9 ± 3.1% and 42.3 ± 6.1% *vs.*73.0 ± 7.2%, respectively, all *P* < 0.01; Figures 2(e) and 2(c)–2(f)).

### 3.2. XKS Improves Endothelial-Mediated Vasodilation in CAD Patients with Anxiety/Depression

**Figure 3:**
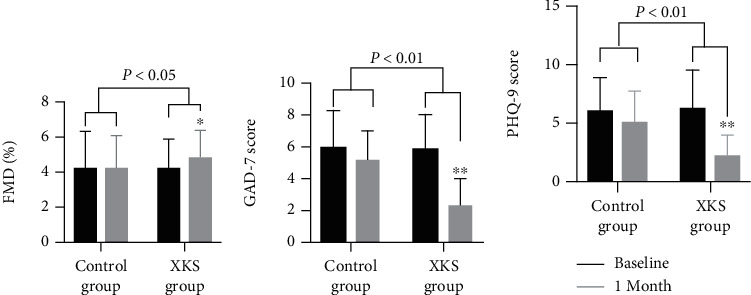
The effects of XKS on FMD, GAD-7, and PHQ-9. (a) The mean value of FMD (^∗^*P* < 0.05*vs.* baseline). (b) The mean value of GAD-7 score (^∗∗^*P* < 0.01*vs.* baseline). (c) The mean value of PHQ-9 score (^∗∗^*P* < 0.01*vs.* baseline).

To investigate the effects of XKS on the endothelial function in CAD patients with anxiety/depression, patients were randomly divided into two groups to receive the XKS intervention or routine treatment. Baseline characteristics were not significantly different between the control and XKS groups (Table S3). It has been demonstrated that FMD is a highly reliable measurement to indicate endothelial function [11]. After 1 month of treatment, FMD changed from 4.29 ± 1.65% to 4.87 ± 1.58% (*P* < 0.05, Figure 3(a)) in the XKS group and 4.28 ± 2.08 to 4.27 ± 1.88% (*P* > 0.05, Figure 3(a)) in the control group, and the XKS group showed significant increase in FMD compared with that in the control group (*P* < 0.05; Figure 3(a)).

### 3.3. XKS Ameliorates GAD-7 and PHQ-9 Score of CAD Patients with Anxiety/Depression

At baseline, patients presented at least a mild level of anxiety or depression symptoms, and the GAD-7 and PHQ-9 scores of the participants were similar between the two groups (Table S3). After 1 month, the average GAD-7 scores in the XKS and control groups were 2.33 ± 1.68 and 5.4 ± 2.06 (*P* < 0.01; Figure 3(b)), respectively. The average PHQ-9 scores were 2.27 ± 1.76 and 5.4 ± 2.82 (*P* < 0.01; Figure 3(c)), respectively. Compared with the control group, anxiety and depression symptoms were improved in patients treated with XKS.

### 3.4. XKS Restores the In Vitro Functions and In Vivo Endothelial Reparative Capacity of EPCs from CAD Patients with Anxiety/Depression

**Figure 4:**
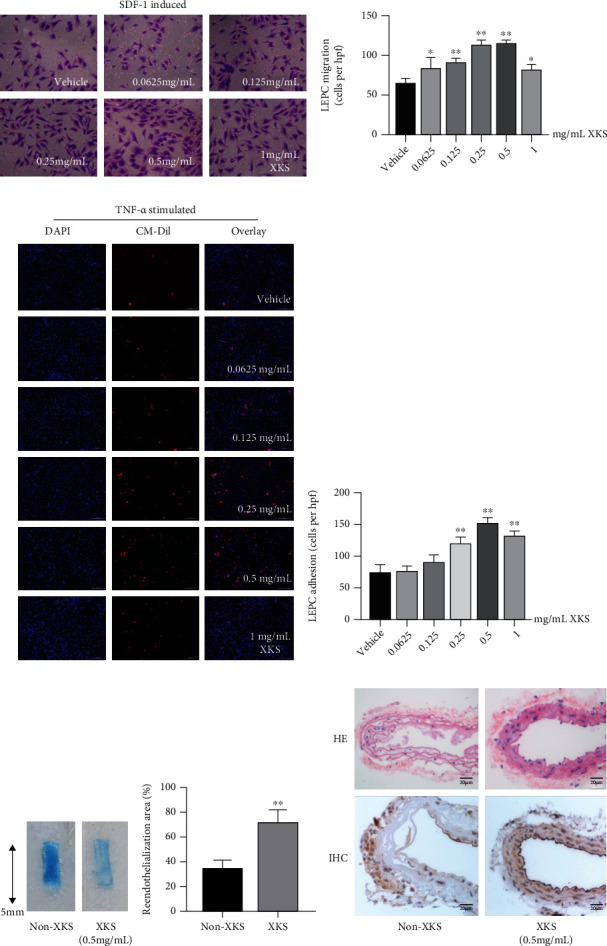
XKS improves the *in vitro* functions and *in vivo* endothelial repair capacity of EPCs. (a) The representative photographs of migration function and (b) the mean value of migrated cell numbers of EPCs incubated among different concentrations of XKS (^∗^*P* < 0.05 and ^∗∗^*P* < 0.01*vs.* the vehicle group, *n* = 6). (c) The representative photographs of adhesion function and (d) the mean value of adhering cell numbers of EPCs incubated among different concentrations of XKS (^∗^*P* < 0.05 and ^∗∗^*P* < 0.01*vs.* the vehicle group, *n* = 6). (e) Representative photographs and quantification analyses of reendothelialization areas (white) of the injured carotid arteries in nude mice 3 days after carotid denudation surgery plus intravenous injection of EPCs (5 × 10^5^ cells) with or without XKS (^∗∗^*P* < 0.01*vs.* the non-XKS group, *n* = 6). (f) The pathological changes of endothelium from carotid arteries in hematoxylin-eosin (HE) and immunohistochemistry (IHC) staining of CD31 (magnification: 400).

EPCs were preincubated with 0.0625, 0.125, 0.25, 0.5, and 1 mg/mL XKS. XKS increased the *in vitro* activities (migration [Figures 4(a) and 4(b)] and adhesion [Figures 4(c) and 4(d)]) of EPCs (*P* < 0.05). According to the increasing extent and *in vitro* activities, we selected 0.5 mg/mL XKS to assess the effect of XKS treatment on EPC-mediated reendothelialization *in vivo*. The EPCs were pretreated with 0.5 mg/mL XKS and then systemically injected into nude mice subjected to carotid artery injury. It was observed that transplantation of *in vitro* XKS-treated EPCs enhanced the *in vivo* reendothelialization area (73.33 ± 8.5% *vs.*34.00 ± 7.0%, *P* < 0.01; Figure 4(e)) and the endothelial integrity presented in HE and IHC (Figure 4(f)) compared to those without XKS treatment.

### 3.5. XKS Enhances CXCR7 Signaling of EPCs from CAD Patients with Anxiety/Depression

**Figure 5:**
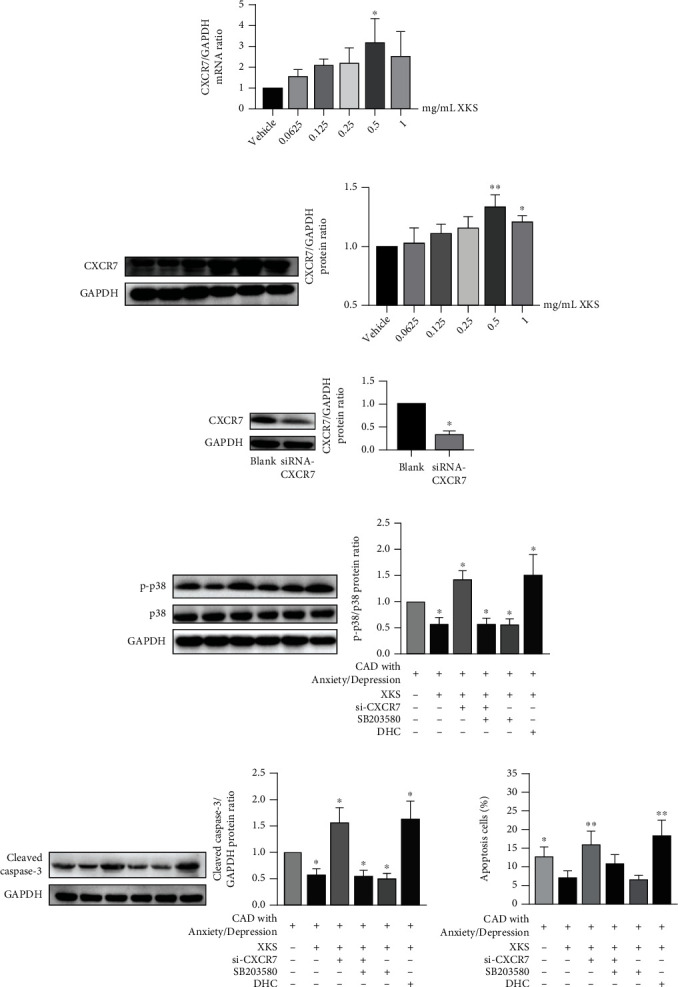
XKS regulates CXCR7/p38/cleaved casepase-3 pathway and inhibits apoptosis of EPCs from CAD patients with anxiety/depression. (a) The quantification analyses of CXCR7 mRNA (^∗^*P* < 0.05*vs.* the vehicle group, *n* = 6). (b) The protein bands of CXCR7 and GAPDH and the mean value of CXCR7/GAPDH ratio (^∗^*P* < 0.05 and ^∗∗^*P* < 0.01*vs.* the vehicle group, *n* = 6). (c) The protein bands of CXCR7 and GAPDH after transfecting with siRNA-CXCR7 and the mean value of CXCR7/GAPDH ratio (^∗^*P* < 0.01*vs.* the non-XKS group, *n* = 6). (d) The protein bands of p-p38, p38, and GAPDH and the mean value of p-p38/p38 ratio (^∗^*P* < 0.01*vs.* the blank group, *n* = 6). (e) The protein bands of cleaved casepase-3 and GAPDH and the mean value of cleaved casepase-3/GAPDH ratio (^∗^*P* < 0.01*vs.* the non-XKS group, *n* = 6). (f) The quantification analyses of apoptosis cells rate measured by flow cytometry (^∗^*P* < 0.01*vs.* the XKS group; *n* = 3 per group).

After preincubation with 0.0625, 0.125, 0.25, 0.5, and 1 mg/mL XKS, we found that *in vitro* XKS treatment led to an increase of CXCR7 level in the EPCs from CAD patients with anxiety/depression, particularly at 0.5 mg/mL (*P* < 0.01) (Figures 5(a) and 5(b)). Moreover, our data revealed that XKS decreased p38 phosphorylation, whereas CXCR7-siRNA blocked the effects of XKS and resulted in an increased p-p38 expression in EPCs, which can be inhibited by p38 inhibitor, SB203580 (*P* < 0.01; Figure 5(d)), while p38 activator dehydrocorydaline (DHC) increased the expression of p-p38 which was reduced by XKS (*P* < 0.01).

### 3.6. XKS Regulates Apoptosis and Cleaved Caspase-3 Expression of EPCs from CAD Patients with Anxiety/Depression

Considering that CXCR7 and p38 regulates EPCs survival [21, 24], we further investigated the effect of XKS on apoptosis and cleaved caspase-3 level of EPCs which was influenced by CXCR7/p38 MAPK signaling. Our data indicated that XKS can reduce apoptosis rate (Figure 5(f)). Meanwhile, siRNA-CXCR7 and DHC markedly attenuated the antiapoptosis capacity of XKS-incubated EPCs and this effect can be inhibited by SB203580 (Figure 5(f)). In parallel, the expression of cleaved caspase-3 was reduced with the incubation of XKS or SB203580 and increased when transfected with siRNA-CXCR7 or incubated DHC (Figure 5(e)).

### 3.7. XKS-Mediated CXCR7 Signaling Enhances the In Vitro Functions and In Vivo Endothelial Repair Capacity of EPCs from CAD Patients with Anxiety/Depression

**Figure 6:**
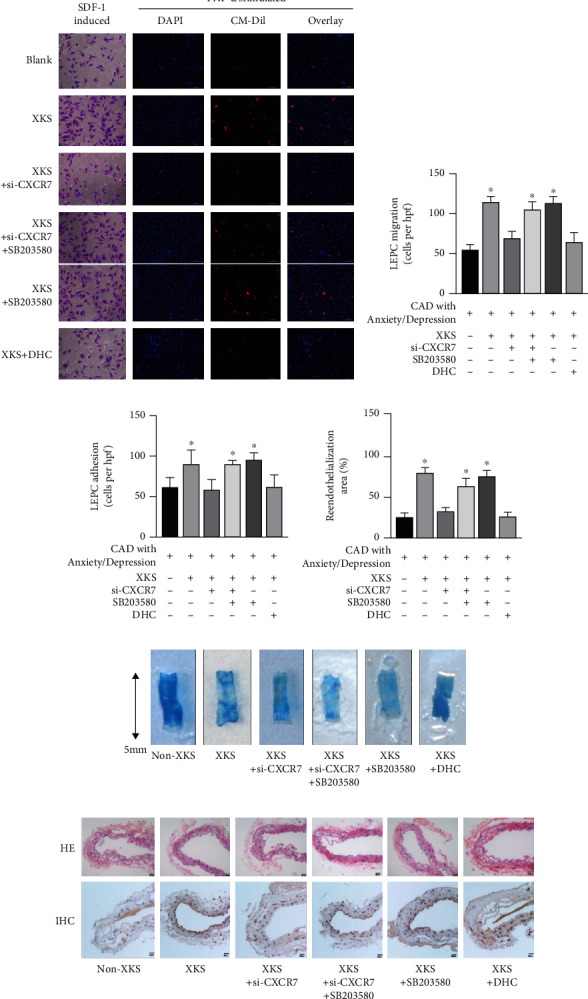
XKS-mediated CXCR7 signaling improves *in vitro* functions and *in vivo* endothelial repair capacity of EPCs. (a) The representative photographs of migration and adhesion function, (b) the mean value of migrated cell numbers (^∗^*P* < 0.01*vs.* the non-XKS group, *n* = 6), and (c) the mean value of adhering cell numbers (^∗^*P* < 0.01*vs.* the non-XKS group, *n* = 6) of EPCs. (d) The quantification analyses and (e) representative photographs of reendothelialization areas (white) of the injured carotid arteries in nude mice 3 days after carotid denudation surgery plus intravenous injection of EPCs (5 × 10^5^ cells) with or without XKS (^∗^*P* < 0.01*vs.* the non-XKS group, *n* = 6). (f) The pathological changes of endothelium from carotid arteries in hematoxylin-eosin (HE) and immunohistochemistry (IHC) staining of CD31 (magnification: 400).

We further investigated the effect of XKS on CXCR7-regulated *in vitro* functions and *in vivo* reendothelialization of EPCs from patients with CAD and anxiety/depression. Our data showed that the increase in the *in vitro* functions of EPCs in the XKS-incubated group was abolished by CXCR7-siRNA transfection (*P* < 0.01); however, it was restored when incubated with the p38 inhibitor SB203580 (Figures 6(a)–6(c)). Consistent with the *in vitro* results, CXCR7-siRNA attenuated the reduced *in vivo* reendothelialization capacity mediated by transplantation of XKS-pretreated EPCs from CAD patients with anxiety/depression (*P* < 0.01; Figures 6(d)–6(f)). Besides, the enhancement of *in vitro* function and *in vivo* reendothelialization capacity of EPCs regulated by XKS was reversed by DHC (*P* < 0.01).

## 4. Discussion

Our present study showed that endothelial function and EPC-mediated endothelium-reparative capacity were significantly impaired in CAD patients with anxiety/depression compared to healthy subjects and even worse than CAD patients without anxiety/depression. XKS treatment improved FMD and ameliorated GAD-7 and PHQ-9 in CAD patients with anxiety/depression symptoms in clinic. Moreover, XKS increased the *in vivo* endothelial repair capacity and *in vitro* function of EPCs derived from CAD patients with anxiety/depression. Further analysis found that enhanced CXCR7 expression and decreased p38 MAPK phosphorylation were associated with these improvements which indicates the important role of CXCR7/p38 MAPK signaling. Besides, XKS-mediated CXCR7/p38 MAPK signaling reduced cleaved caspase-3-regulated antiapoptosis functions of EPCs from CAD patients with anxiety/depression. To the best of our knowledge, this is the first study aimed at elucidating whether XKS exerts protective effects on endothelial function and endothelium-reparative capacity in CAD patients with anxiety/depression, and the role of CXCR7/p38/cleaved casepase-3 signaling pathway in XKS-regulated reendothelialization capacity.

Accumulating evidence shows that psychological factors such as anxiety and depression are strongly associated with the occurrence and progression of CAD [1, 2]. A meta-analysis concluded that anxious population presented a high risk of CAD and cardiac death, independent of the demographic variables, biological risk factors, and health behaviors [29]. Presently, due to several adverse effects, the usage of antidepressants is limited in patients with CAD. Previous studies demonstrated that XKS can not only effectively promote the recovery of cardiac function after myocardial ischemia and reperfusion injury [16] but also ameliorate anxiety and depression [17]. In the current study, our data revealed that XKS reduced the GAD-7 and PHQ-9 scores of CAD patients with anxiety/depression symptoms, which is consistent with previous research [17].

Endothelial dysfunction is a major pathology factor that contributes to the poor prognosis of both CAD and anxiety/depression patients. Chen et al. [14] reported that stable angina patients manifest as severe anxiety and depression symptoms than counterparts, which was related with a decreased FMD. EPCs play a crucial role in endogenous injured endothelial recovery and directly participate in vascular repair and promote angiogenesis [12, 13]. Moreover, the declined function and number of circulating EPCs were observed in patients with CAD with anxiety/depression [14, 15]. Our results also showed that endothelial function and EPC-mediated endothelium-reparative capacity were significantly impaired in CAD patients with anxiety/depression compared to healthy subjects and even worse than CAD patients without anxiety/depression. Notably, it has been reported that XKS increases the eNOS level and endothelia-dependent vascular relax in rabbits [16]; however, the effect of XKS on endothelial function and repair of endothelial injury in CAD patients with anxiety/depression was still unknown. Our results indicate that XKS not only improved FMD in CAD patients with anxiety/depression but also upregulated the EPC-mediated reendothelialization capacity in *in vivo* and *in vitro* studies. However, the underlying molecular mechanism remained to be further elucidated.

Emerging data suggest that CXCR7 is essential for regulating cellular biological functions [21, 30, 31]. For instance, evidence shows that CXCR7 protects human renal progenitor cells from apoptosis challenges and regulates renal progenitor cell survival [21]. And recent investigations demonstrated that CXCR7 is an important signaling molecule and plays a pivotal role in the regulation of adhesion and survival functions of in vitro EPCs from both rats and normal human beings [31, 32]. Furthermore, as one of the best studied MAPKs, p38 MAPK was found to influence a multitude of cellular events, such as cell growth and death and cell proliferation [33, 34]. Increasing evidence indicates that p38 MAPK could be part of an important signaling pathway that mediates behavioral changes in depression and anxiety [35–38]. Studies have reported that lipopolysaccharide (LPS) induces the activation of p38, which is associated with the occurrence of depressive-like behaviors, and downregulation of p-p38 by its inhibitor, SB203580, could significantly alleviate depressive symptoms, suggesting that p38 activation is a key process in the induction of depressive-like behaviors [33]. Our previous study revealed that the abnormalities of CXCR7/p38 MAPK signaling pathway led to reduced reendothelialization capacity of EPCs derived from hypertensive patients, and the upregulation of CXCR7 and inhibition of the phosphorylation of p38 MAPK prevent EPC apoptosis and improve endothelial repair capacity [24]. It has also been proved that XKS improves endothelial diastolic function of myocardial ischemia animal model and protects vascular endothelium [16]. As it was found that XKS improved endothelial diastolic function and EPC-mediated reendothelialization capacity in CAD patients with anxiety/depression in this study, we continued to demonstrate that XKS facilitates reendothelialization *in vivo* and augments EPC function *in vitro* via the regulation of CXCR7/p38 signaling which meanwhile could reduce cleaved caspase-3-regulated apoptosis of EPCs. This study may provide the novel insight into the therapeutic target for our understanding of XKS which ameliorates endothelial dysfunction in CAD patients with anxiety/depression.

The present study has certain limitations. First, our study is an open-label clinical trial with a relatively small sample size. For better illustration of the function of XKS, a randomized placebo-controlled trial with large sample size needs to be conducted in the future. Second, we found that XKS induced the regulation of CXCR7/p38/cleaved caspase-3 signaling contributes to the improvement of EPC-mediated endothelial reendothelialization in CAD with anxiety/depression; however, whether XKS exerts its beneficial effect on neuronal cells or other cells via different mechanisms still remains unclear.

## 5. Conclusions

In summary, the present study demonstrated that XKS improves endothelial function and anxiety/depression symptoms in CAD patients with anxiety/depression. Moreover, XKS increases the *in vivo* reendothelialization capacity and *in vitro* activities of EPCs, which were mediated by CXCR7/p38/cleaved casepase-3 signaling. This study provides novel insight into the understanding of the mechanisms of endothelial repair in CAD patients with anxiety/depression. Our results indicate that XKS is an effective pharmacologic intervention to maintain endothelial homeostasis in CAD patients with anxiety/depression.

## Data Availability

The data used to support the findings of this study are available from the corresponding author upon request.

## Supplementary Materials

Supplementary Materials This paper includes supplementary figures and tables (Supplement) which are available online.
