# Supplementary material for: Xinkeshu Improves Endothelial Function and Augments Reendothelialization Capacity in Coronary Artery Disease with Anxiety/Depression
Source: Oxid Med Cell Longev. 2021 Jul 18;2021:5561272. doi: 10.1155/2021/5561272 (PMC8313340; doi:10.1155/2021/5561272)
Supplement: Supplementary Materials — This paper includes supplementary figures and tables (Supplement) which are available online. [file 5561272.f1.docx]

**Online Supplement**

Simultaneous identification and quantification of multi-constituent of XKS was testes by liquid chromatography coupled with electrospray ionization hybridlinear trap quadrupole orbitrap (LC–LTQ-Orbitrap) mass spectrometry to ensure the quality. A total of 51 compounds, including phenolic acids, isoflavone-C-glycosides, isoflavone-O-glycosides, flavonoids, and triterpenoid saponins, were identified or tentatively deduced on the base of their retention behaviors, MS and MSn data, or by comparing with reference substances and literatures. As is shown in Supplemental Figure S1, Supplemental Table S1[1].

**Supplemental Figures and Figure Legends**

**Supplementary Figure S1**


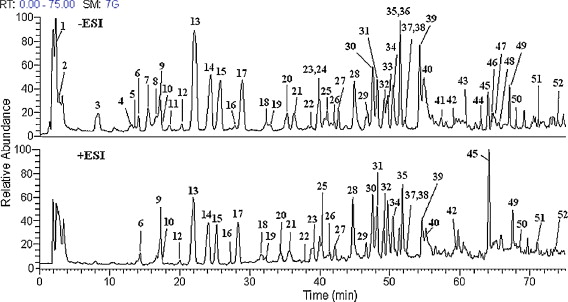


**Figure S1. TIC chromatograms of XKS extract in negative and positive ionization modes [1].**

﻿

**Supplementary Figure S2**

**
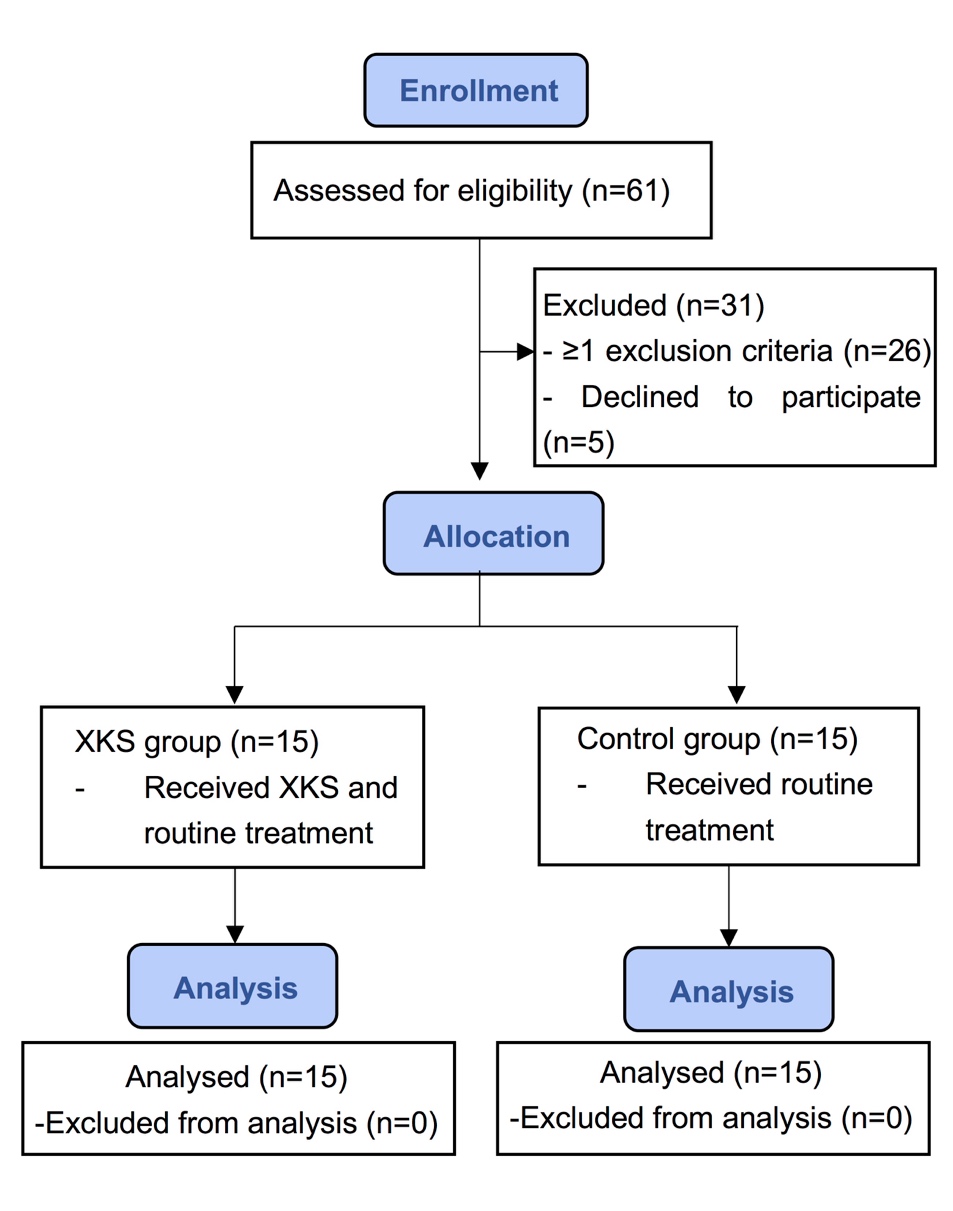
**

**Figure S2. Clinical Trial profile.** Patients were randomly divided into XKS group and control group. 15 patients completed the study in XKS group, 15 patients completed the study in control group.

**Supplementary Figure S3**

**
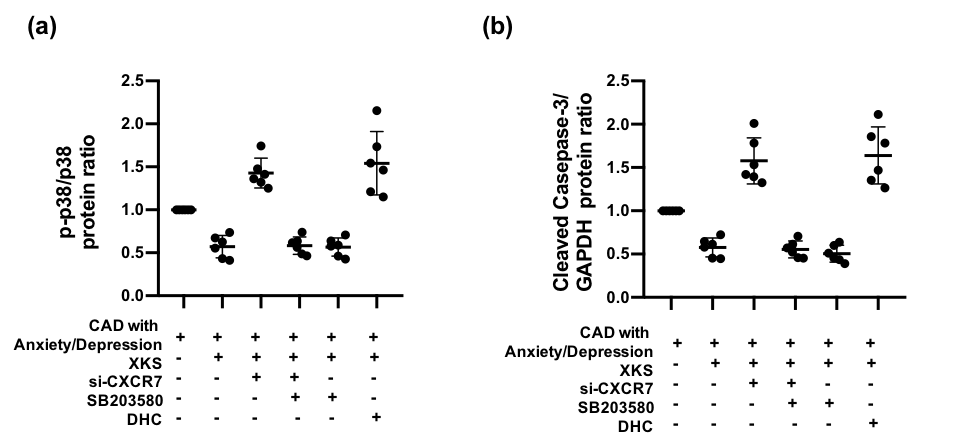
**

**Figure S3. The effects of XKS on p38 and cleaved caspase-3 level.** (a) The scatter plot of p-p38/p38 protein ratio (n = 6). (b) The scatter plot of cleaved casepase-3/GAPDH protein ratio ( n = 6).

**Supplemental Table**

**Supplemental Table S1. Identification of the chemical constituents of XKS preparation by LC–ESI-MSn analysis**


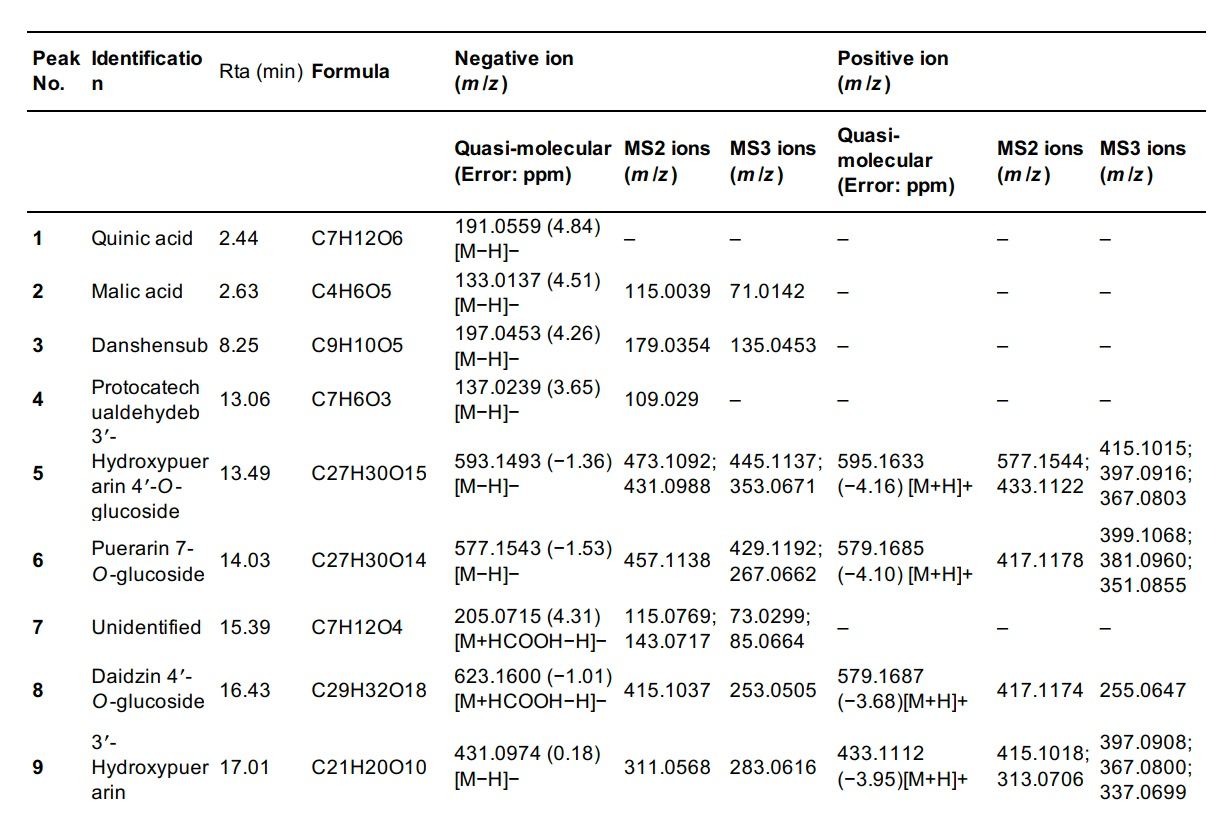


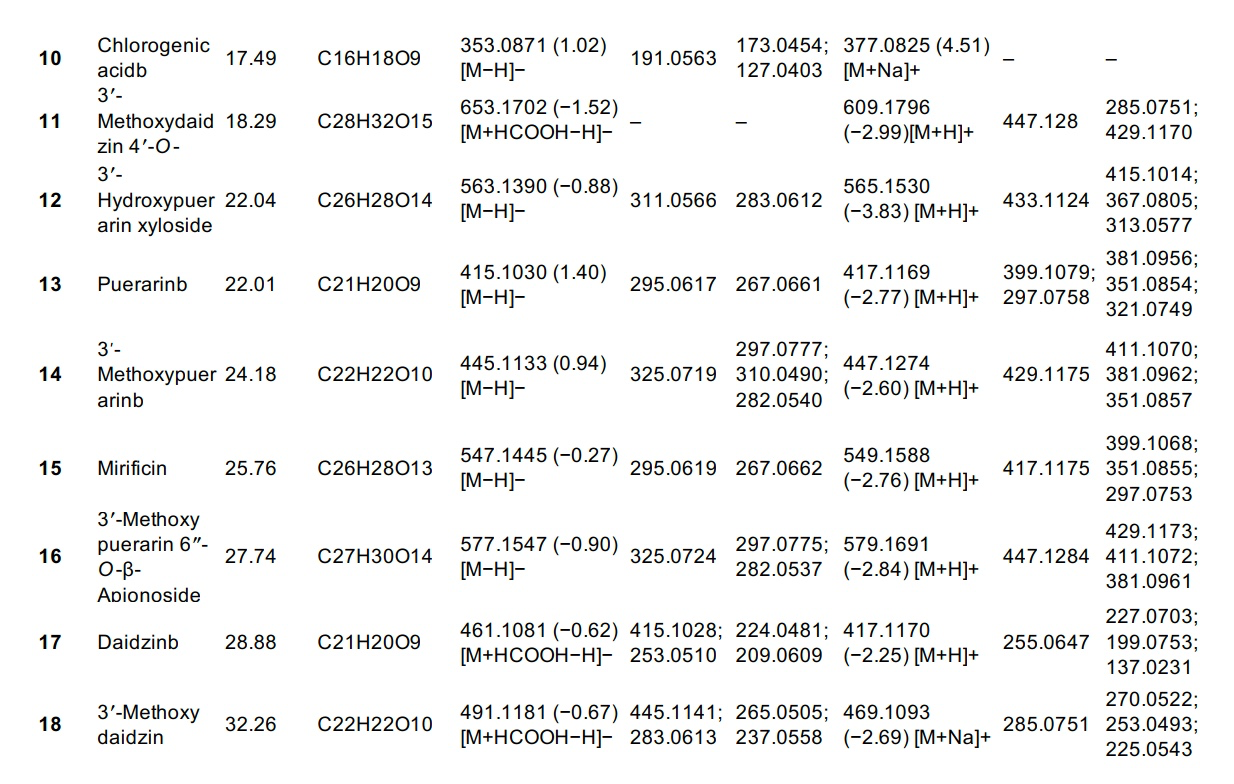


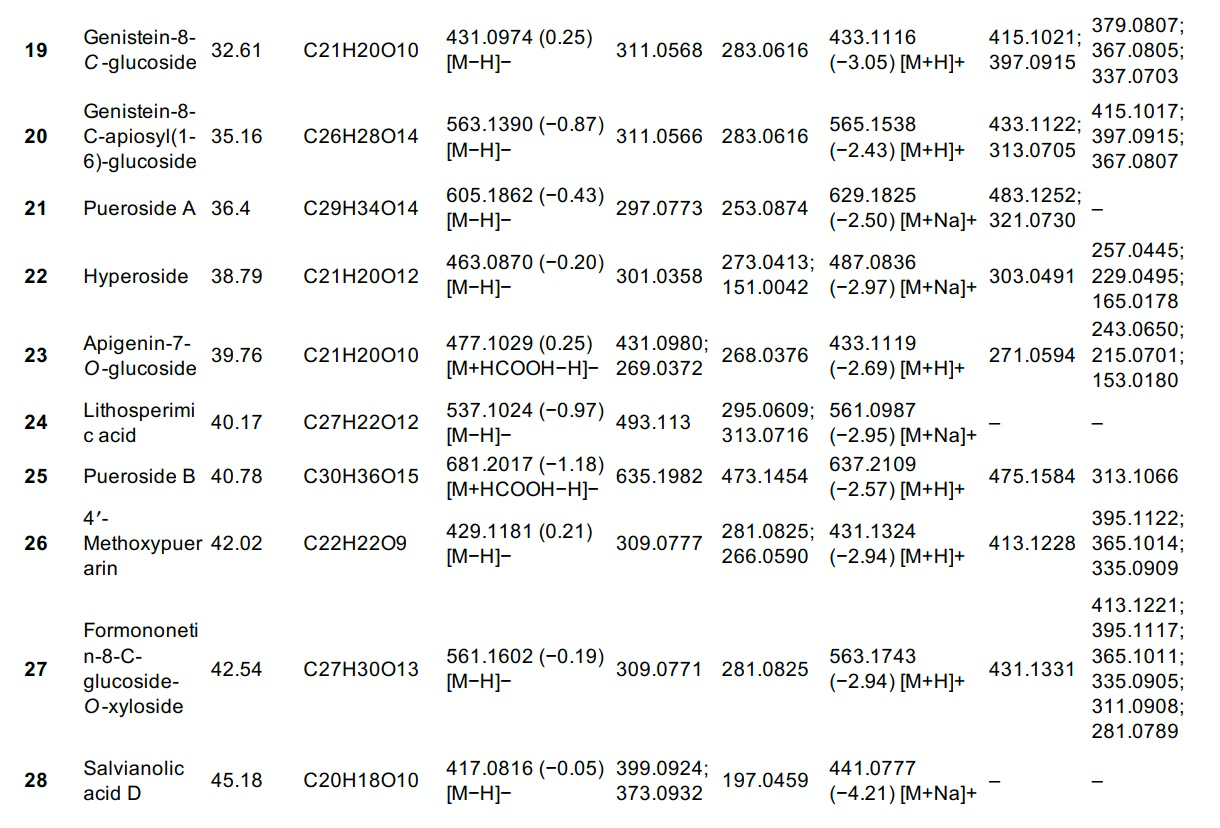


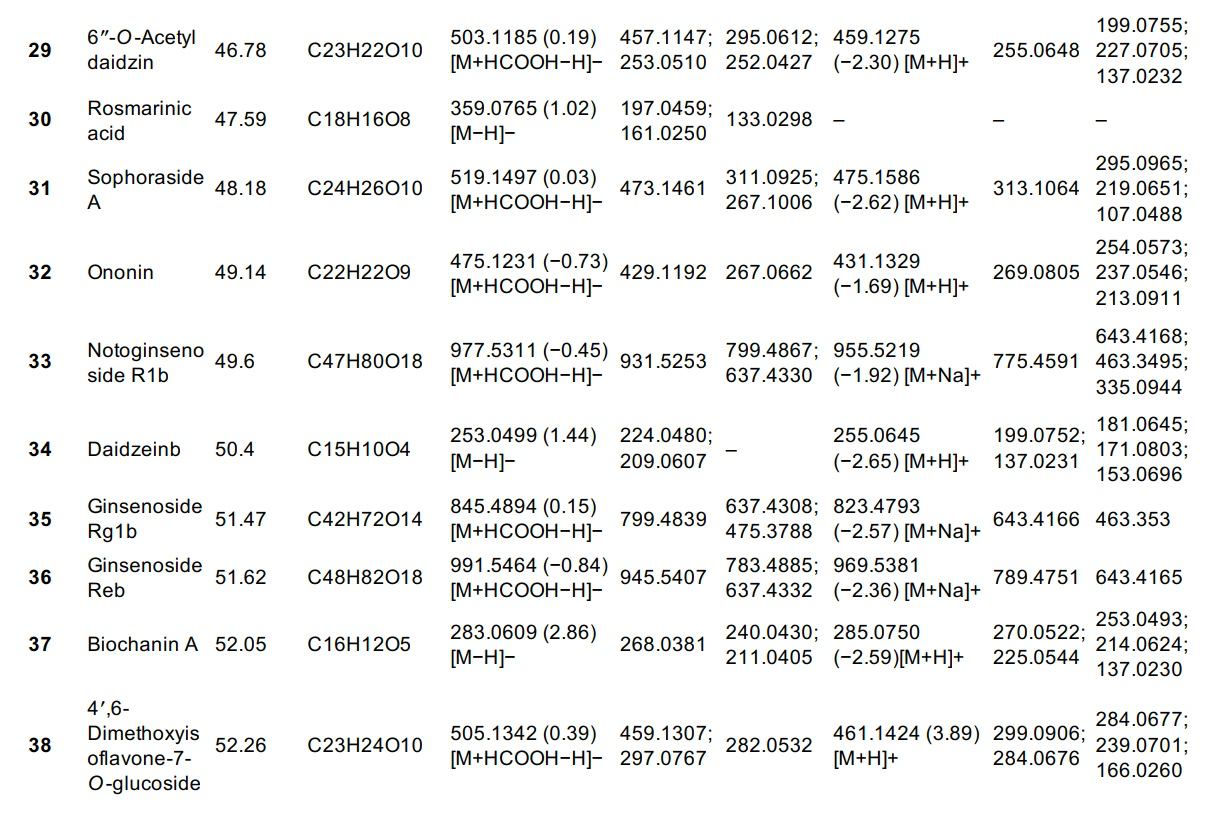


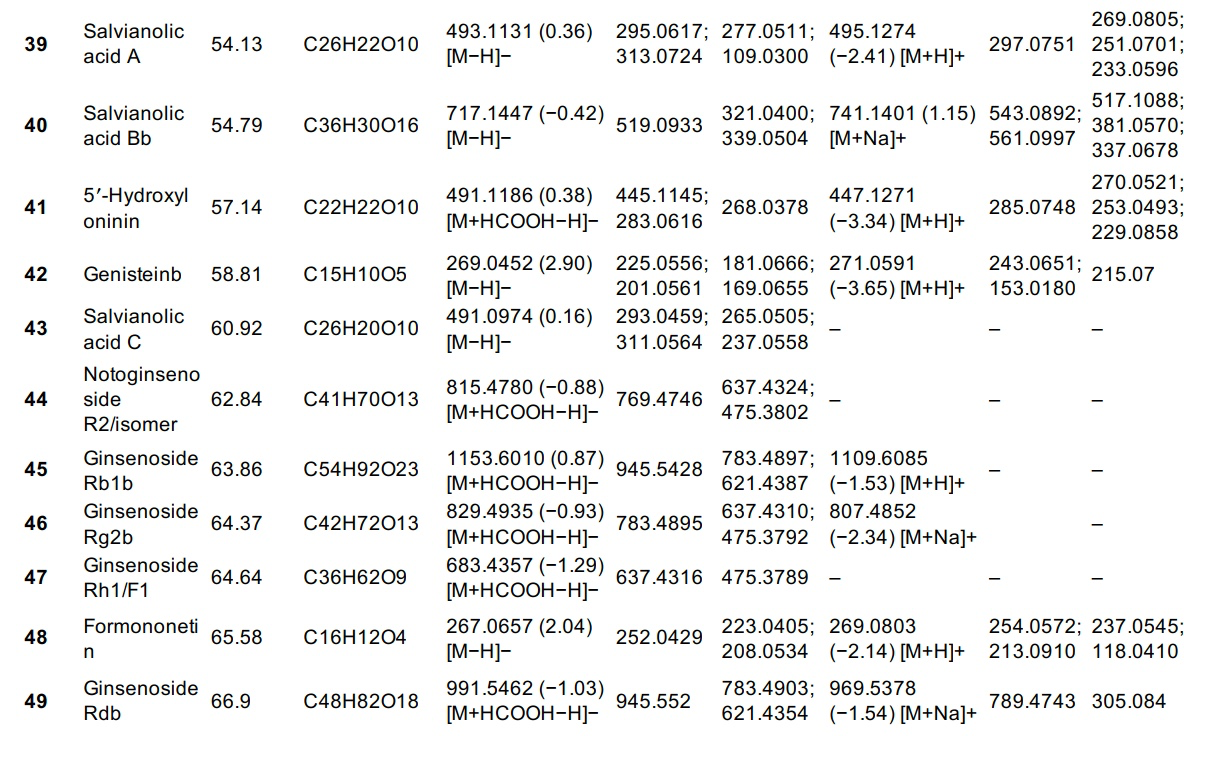

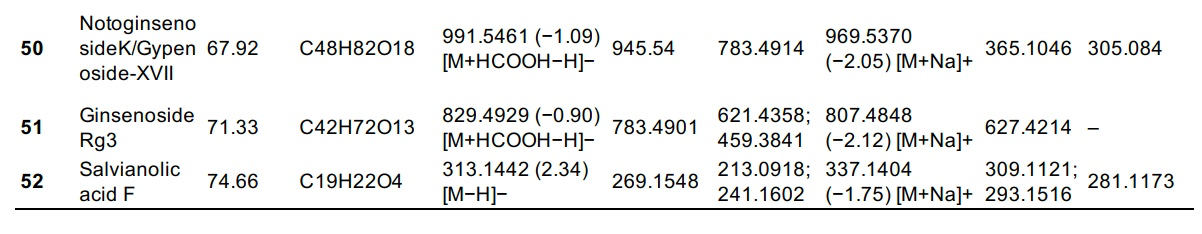


﻿a Rt: retention time.

b Structurally confirmed by comparison with reference chemicals [1].

**Supplemental Table S2. Subjects’ Characteristics**

| **Subject Characteristics** | **CAD Patients with Anxiety/Depression** | **CAD Patients without Anxiety/Depression** | **Healthy Subjects** | ***P* Value** |
| --- | --- | --- | --- | --- |
| Age, y | 55.30 ± 9.23 | 57.5±8.32 | 53.95 ±8.24 | 0.278 |
| Male, n | 17 | 19 | 17 | 0.832 |
| BMI, kg/m2 | 24.38 ± 2.23 | 24.42 ± 2.08 | 23.72 ± 3.21 | 0.495 |
| SBP, mmHg | 137.25 ± 12.91 | 132.32 ± 10.21 | 122.15 ± 9.07 | <0.001^**^ |
| DBP, mmHg | 80.12 ± 9.28 | 82.13 ± 8.88 | 79.21 ± 6.92 | 0.393 |
| FPG, mmol/L | 5.47 ± 0.48 | 5.23±0.59 | 5.39 ± 0.78 | 0.327 |
| TC, mmol/L | 4.43 ± 0.92 | 4.24 ± 1.02 | 4.37 ± 0.79 | 0.714 |
| TG, mmol/L | 1.33 ± 0.42 | 1.42 ± 0.57 | 1.26 ± 0.67 | 0.546 |
| LDL-C, mmol/L | 2.52 ± 0.57 | 2.63 ± 0.64 | 2.61 ± 0.82 | 0.803 |
| HDL-C, mmol/L | 1.09 ± 0.32 | 1.23 ± 0.38 | 1.35 ± 0.52 | 0.057 |

Data are shown as mean ± SD.^*^*P* < 0.05 vs. control group, ^**^*P* < 0.01 vs. control group. CAD = cardiovascular artery disease; BMI = body mass index; SBP = systolic blood pressure; DBP = diastolic blood pressure; FPG = fasting plasma glucose; TC = fasting plasma glucose; TG = triglycerides; HDL-c = high density lipoprotein cholesterol; LDL-c = low-density lipoprotein cholesterol; FMD = flow mediated dilation;

**Supplemental Table S3. Baseline Characteristics of XKS Group and Control Group**

| **Subject Characteristics** | **XKS Group** | **Control Group** | ***P* Value** |
| --- | --- | --- | --- |
| Age, y | 59.87 ± 8.54 | 55.47 ± 10.72 | 0.224 |
| Male, n | 8 | 9 | 0.713 |
| BMI, kg/m2 | 24.05 ± 2.44 | 24.31 ± 2.13 | 0.763 |
| SBP, mmHg | 136.07 ± 17.61 | 139.00 ± 13.28 | 0.610 |
| DBP, mmHg | 77.47 ± 8.22 | 82.67 ± 9.40 | 0.118 |
| FPG, mmol/L | 5.41 ± 1.84 | 5.07 ± 1.35 | 0.325 |
| TC, mmol/L | 4.03 ± 0.60 | 4.57 ± 1.48 | 0.285 |
| TG, mmol/L | 1.18 ± 0.54 | 1.63 ± 0.90 | 0.174 |
| LDL-C, mmol/L | 2.41 ± 0.40 | 2.73 ± 0.87 | 0.305 |
| HDL-C, mmol/L | 1.13 ± 0.21 | 1.20 ± 0.49 | 0.967 |
| FMD, % | 4.29 ± 1.65 | 4.28 ± 2.08 | 0.992 |
| GAD-7 | 5.93 ± 2.12 | 6.00 ± 2.30 | 0.935 |
| PHQ-9 | 6.40 ±3.23 | 6.13 ± 2.85 | 0.812 |

Data are shown as mean ± SD.^*^*P* < 0.05 vs. control group, ^**^*P* < 0.01 vs. control group. CAD = cardiovascular artery disease; BMI = body mass index; SBP = systolic blood pressure; DBP = diastolic blood pressure; FPG = fasting plasma glucose; TC = fasting plasma glucose; TG = triglycerides; HDL-c = high density lipoprotein cholesterol; LDL-c = low-density lipoprotein cholesterol; FMD = flow mediated dilation; GAD-7 = Generalized Anxiety Disorder 7-item ; PHQ-9 = Patient Health Questionnaire-9.

**﻿**

**Supplemental References**

[1] J. Peng, H. Jia, Y. Liu, et al., “Qualitative and quantitative characterization of chemical constituents in Xin-Ke-Shu preparations by liquid chromatography coupled with a LTQ Orbitrap mass spectrometer,” *Journal of pharmaceutical and biomedical analysis*, vol. 55, no. 5, pp. 984-995, 2011.
